# In vitro evaluation of dye penetration and dentin microhardness after laser irradiation using photon-induced photoacoustic streaming and shock wave enhanced emission photoacoustic streaming tips compared to ultrasonic activation

**DOI:** 10.1007/s10103-025-04310-4

**Published:** 2025-01-31

**Authors:** Ahmed Moustafa Mobarak, Yomna Mohamed Ibrahim, Ahmed Adel Abdelhakim, Josep Arnabat-Dominguez, Rania Mahmoud El Backly

**Affiliations:** 1 Conservative Dentistry Department, Faculty of Dentistry, Alexandria University, Alexandria, Alexandria, Egypt; 2 Dental Biomaterials Department, Faculty of Dentistry, Alexandria University, Alexandria, Alexandria, Egypt; 3 Department of Prosthodontics, Faculty of Dentistry, Alexandria University, Alexandria, Alexandria, Egypt; 4 Faculty of Medicine and Health Sciences, Dental school, University of Barcelona, Investigator of the IDIBELL Institute, Barcelona, Barcelona, Spain; 5 Conservative Dentistry Department and Tissue Engineering Laboratories, Faculty of Dentistry, Alexandria University, Alexandria, Alexandria, Egypt

**Keywords:** Er:YAG laser, PIPS, SWEEPS, PUI, Dye penetration, Microhardness

## Abstract

The purpose of this study was to compare the penetration of methylene blue (MB) dye after laser irradiation using PIPS (photon-induced photoacoustic streaming) and SWEEPS (shockwave enhanced emission photoacoustic streaming) methods compared to Passive ultrasonic irrigation (PUI) and to study their effect on dentin microhardness. A total of 44 single-rooted human teeth which were extracted for orthodontic or periodontal reasons were used. The teeth were decapitated to standardize roots to 12 mm in length. Canals were prepared up to size #30.6% and divided randomly into four groups (*n* = 11/group) according to the method of NaOCl activation: Group I: Er: YAG laser activation with PIPS tip; Group II: Er: YAG laser activation with SWEEPS tip; Group III: passive ultrasonic irrigation (PUI) and Group IV: conventional needle irrigation (CI). MB dye was injected then teeth were sectioned horizontally into coronal, middle and apical sections. Penetration depth/area percentages were measured using stereomicroscopy. Coronal specimens were further subjected to Vickers microhardness testing. Data were statistically analyzed. SWEEPS and PIPS activation methods provided higher dye penetration depth and area percentages compared to PUI with no statistically significant differences between all test groups. However, all test groups showed statistically significant differences with the CI (control) group. SWEEPS activation provided higher microhardness values with statistically significant differences with the other groups. Laser irrigant activation using PIPS and SWEEPS is comparable to PUI concerning MB dye penetration. However, SWEEPS preserved dentin microhardness significantly which can be beneficial for the long-term prognosis of root canal treated teeth.

**Supplementary Information:**

The online version contains supplementary material available at 10.1007/s10103-025-04310-4.

## Background

The main goal of endodontic treatment is the removal of infected pulpal tissues, eradication of bacteria found inside the dentinal tubules (DTs) and root canals, and prevention of re-contamination after treatment using combination of chemical irrigation and mechanical instrumentation [1].

During endodontic therapy, effective irrigation is an essential step for the successful removal of bacteria and debris from the root canal system. The depth of penetration of irrigants inside the DTs is an important factor to consider, as it determines the extent to which the root canal system is cleaned and disinfected [2]. Literature has shown that root canal irrigants can penetrate only a few micrometers into the DTs, leaving the deeper part of DTs untouched where microbial biofilms may be deeply situated [2, 3].

To overcome this limitation, several strategies and techniques have been developed to enhance the penetration depth of irrigants, including; manual dynamic agitation, thermal activation of irrigants [4], and mechanical techniques such as ultrasonic and sonic activation, creating acoustic streaming and cavitation pushing irrigants into deeper portions of the DTs [5].

The use of lasers for irrigant activation, has been shown to be a potentially valuable aid to facilitate smear layer removal during endodontic treatment and enhance irrigant penetration inside DTs [6]. Er: YAG lasers (2940 nm wavelength) have been approved by the FDA for cleaning and shaping of root canals [7]. Also, using Er: YAG lasers is an effective way that can be used for smear layer removal, exposing the DTs, thus allowing irrigants to penetrate more deeply inside the tubules [8].

Recently, a new technique for the use of Er: YAG lasers has been introduced namely Photon-induced Photoacoustic Streaming (PIPS) which has been shown to have a positive radial effect on the removal of debris and smear layer, leading to an increased rate of cleaning of the canal walls when compared to other conventional approaches [9]. The other proposed method is called Shockwave-Enhanced Emission Photoacoustic Streaming (SWEEPS) to specifically enhance the cleaning and disinfecting efficacy of endodontic treatments [10]. By means of this technique, a series of bubbles are formed that in time are replaced with secondary bubbles and lead to shockwave formation and an improved photoacoustic current [3]. Another potential advantage of these newly introduced techniques is their ability to reduce the deleterious effects of irrigants on dentin microhardness [11]. This is of particular interest since the use of irrigants and their activation may reduce the microhardness, fracture resistance and elastic modulus of the dentin. Moreover, prolonged exposure to irrigant agents may increase susceptibility to vertical root fracture [12]. Although the effect of endodontic irrigants on dentin microhardness have been extensively investigated, the effect of irrigants varies according to the activation mode remains controversial [13, 14]. While these novel techniques, PIPS and SWEEPS, may have possible advantages to improve irrigant penetration, there have been very limited studies assessing their effects in clinical settings and results of in vitro studies have been controversial [15].

Accordingly, the present study aimed to determine whether there would be a difference in the penetration depth of methylene blue dye inside the DTs after laser irradiation using PIPS and SWEEPS methods compared to PUI or CI and to study the effect of these activation methods on dentin microhardness. The null hypothesis tested was that there would be no difference among the final irrigation activation techniques in terms of dye pentration depth/area % and dentin microhardness values.

## Methods

This study was conducted at the Laser facility of the Faculty of dentistry, Alexandria University after the approval of the Research Ethics Committee of Alexandria University, Faculty of Dentistry, Egypt (IRB No. 001056– IORG 0008839) (Serial number: 0732-07-2023 in 30/7/2023). The minimum sample size for each group was determined at 11 by using the results of Kosarieh et al.’s study [16] using a one-way ANOVA power analysis option in PASS11 software considering *p* < 0.05 and 95% confidence level [17].

A total of 44 extracted human permanent single-rooted teeth with uncalcified single root canals with completely formed roots teeth which were extracted for orthodontic or periodontal reasons from patients ranged from 18 to 50 years old were selected for this study. All teeth were cleaned by washing under running water and were immersed in 5.25% sodium hypochlorite for 30 min to remove soft tissue attached to the root surface. Further cleaning was done using an ultrasonic scaler to remove any remaining calculus or soft tissue. Teeth were then stored in 0.1% thymol solution (Sigma-Aldrich, St. Louis, MO, USA), then they were placed in saline solution until use [15].

Samples were decoronated under water coolant by a low-speed diamond saw (Isomet Low- Speed Saw, Buehler, Lake Bluff, IL, USA), so that the remaining apical parts were standardized at 12 mm long. The working length was determined by placing a #15 k-file (MANI, INC, Tokyo, Japan) inside the canal and when the file was observed at the end of the canal, 0.5 mm was subtracted from that length and set as the working length. Two coatings of nail polish were applied to the external surfaces to prevent contamination, also the apical foramina were sealed with composite resin (ESTELITE ALPHA, Tokuyama Dental Corporation, Tokyo, Japan).

The root canals were instrumented using the crown-down technique with endodontic rotary files up to master apical file #30/6% (EdgeEndo, Albuquerque, NM, USA). NaOCl (2.5%) was used for irrigation during the instrumentation step using a 30-gauge side-vented needle (EndoTop, Cerkamed, Kwiatkowskiego, Poland) placed 1 mm short of the working length. Root canals were then rinsed with 10 mL of distilled water and, subsequently, irrigated with 3 ml 17% EDTA solution and left in the canals for 1 min before being rinsed again with 10 mL distilled water and, subsequently, with 3 mL 2.5% NaOCl. Specimens were again rinsed with 10 mL of distilled water. Root canals were finally dried using paper points (META BIOMED, Chungcheongbuk-do, Republic of Korea) [9].

Samples were then randomly divided by using a computer-generated list of random numbers (www.randomizer.org) into four groups according to the method of post-shaping irrigant activation procedures.

In group I (*n* = 11), laser irradiations were done using Er: YAG laser device (Light Walker, Fotona, Ljubljana, Slovenia) with 2940 nm wavelength. The manufacturer protocol for the PIPS technique (50µs, SSP, 15 Hz, 20mj, 0.3 W) was used using an H14 handpiece and PIPS tip (0.6 mm/9 mm, cylindrical, tapered). The air and water on the laser system were switched off [15]. For group II (*n* = 11), the Er: YAG laser device with 2940 nm wavelength using the H14 handpiece and SWEEPS tip (0.6 mm/9 mm, cylindrical, tapered) with the protocol for the SWEEPS technique (50µs, SWEEPS mode, 15 Hz, 20mj, 0.3 W) as recommended by the manufacturer [15]. Regarding groups I and II; PIPS and SWEEPS tips were placed only in the coronal part of the canals (2–3 mm) which were flooded with 2.5% NaOCl and activated for three cycles of 20 s each, so that each canal was subjected to 1 min of laser activation [18]. For group III (*n* = 11), root canals were irrigated with 2.5% NaOCl then the ultrasonic tip (ED93, Woodpecker, Guangxi, China) was placed till 1 mm short of the working length. The ultrasonic tip was kept centered in the canal and 2–3 mm apical-coronal movements were done for 20 s. The passive irrigation technique with an intermittent flush consisted of applying 3 cycles of ultrasonic activation of the irrigant for 20 s each, so that each canal was subjected to 1 min of ultrasonic activation [19]. For the group IV (control) (*n* = 11); all teeth were irrigated with 2.5% NaOCl for 1 min using a 30-gauge side-vented irrigation needle with no additional method of activation.

The root canals were then dried using absorbent paper points then 1% methylene blue dye (Sigma-Aldrich Pty Ltd, Missouri, United States) was injected into prepared root canals using a 30-gauge side-vented irrigation needle and the dye was left inside the canals for 10 min. After that, final drying of the root canals was done using absorbent paper points [15].

### Teeth sectioning and dye penetration depth/area calculation

Cross sections of the teeth were prepared using a circular saw (Leitz 1600, Ernst Leitz Wetzlar, Wetzlar, Germany) under constant water flow to divide each tooth to coronal, middle and apical thirds (4 mm each). The thirds of each tooth were imaged at x35 using a stereomicroscope (B061, Olympus, Japan). Fiji software (ImageJ, version 2.14.0, NIH, USA) was used to measure the penetration depth and the area covered by methylene blue dye. To measure the penetration depth, a virtual clock was placed over the image in the center of the canal and 24 lines were extended to measure dye penetration in micrometers [3]. This measurement was divided by dentin thickness to calculate dye penetration as a percentage of the section radius. The area of dye penetration was calculated using the color threshold feature by confining the color to blue then transforming it to black using the binary option and the black area was measured in micrometers squared. This area was divided by total dentin area to calculate percentage of dye coverage in relation to total section area [20].

### Vickers microhardness test

To measure the dentin microhardness, the coronal third was mounted on the stage of a Vickers microhardness tester (HVS-1000 A; Jinan Hensgrand Instrument Co., Ltd.) and a load of 300 g was applied for 20 s to make an indentation in dentin 200 micrometers from the dentin/canal interface. The diagonals of the indentation were measured, and the Vickers hardness number (VHN) was calculated then the average of three indentations was calculated for the coronal thirds of each group [21].

### Statistical analysis

Origin software (OriginPro, Version 2024, OriginLab Corporation, USA) was used for statistical analysis. Data normality was assessed using Shapiro-Wilk test and Q-Q plots. All variables were normally distributed and thus represented by means and standard deviation. Two-way ANOVA was used for penetration depth % and penetration area % assessment, while One-way ANOVA was used for Vickers microhardness assessment. Tukey post hoc test was used with subsequent Bonferroni correction (α = 0.05) for pairwise comparisons among study groups and tooth thirds.

## Results

**Fig. 1:**
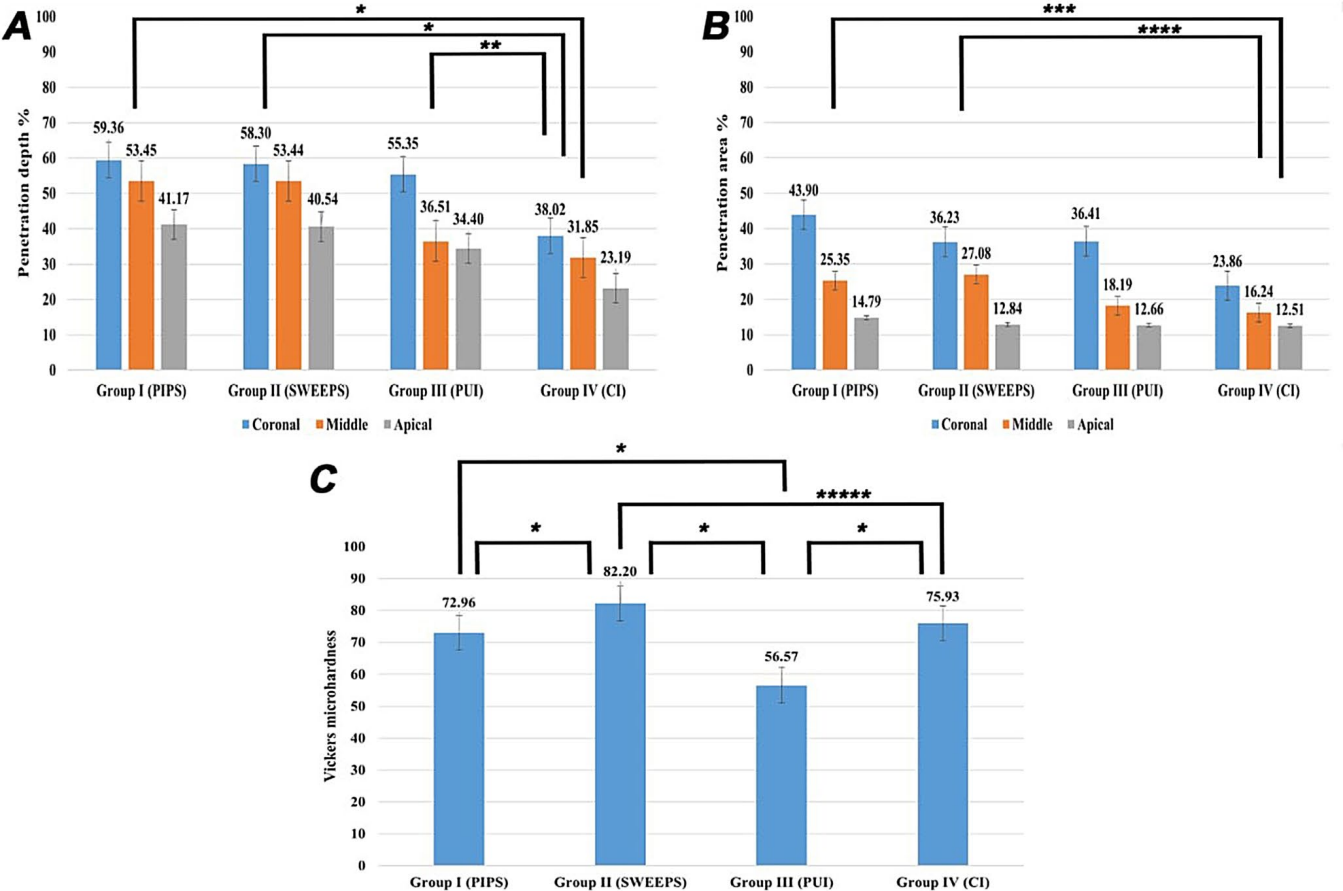
Comparisons among groups regarding (**A**) Penetration depth %, (**B**) Penetration area %, and (**C**) Vickers microhardness values

**Fig. 2:**
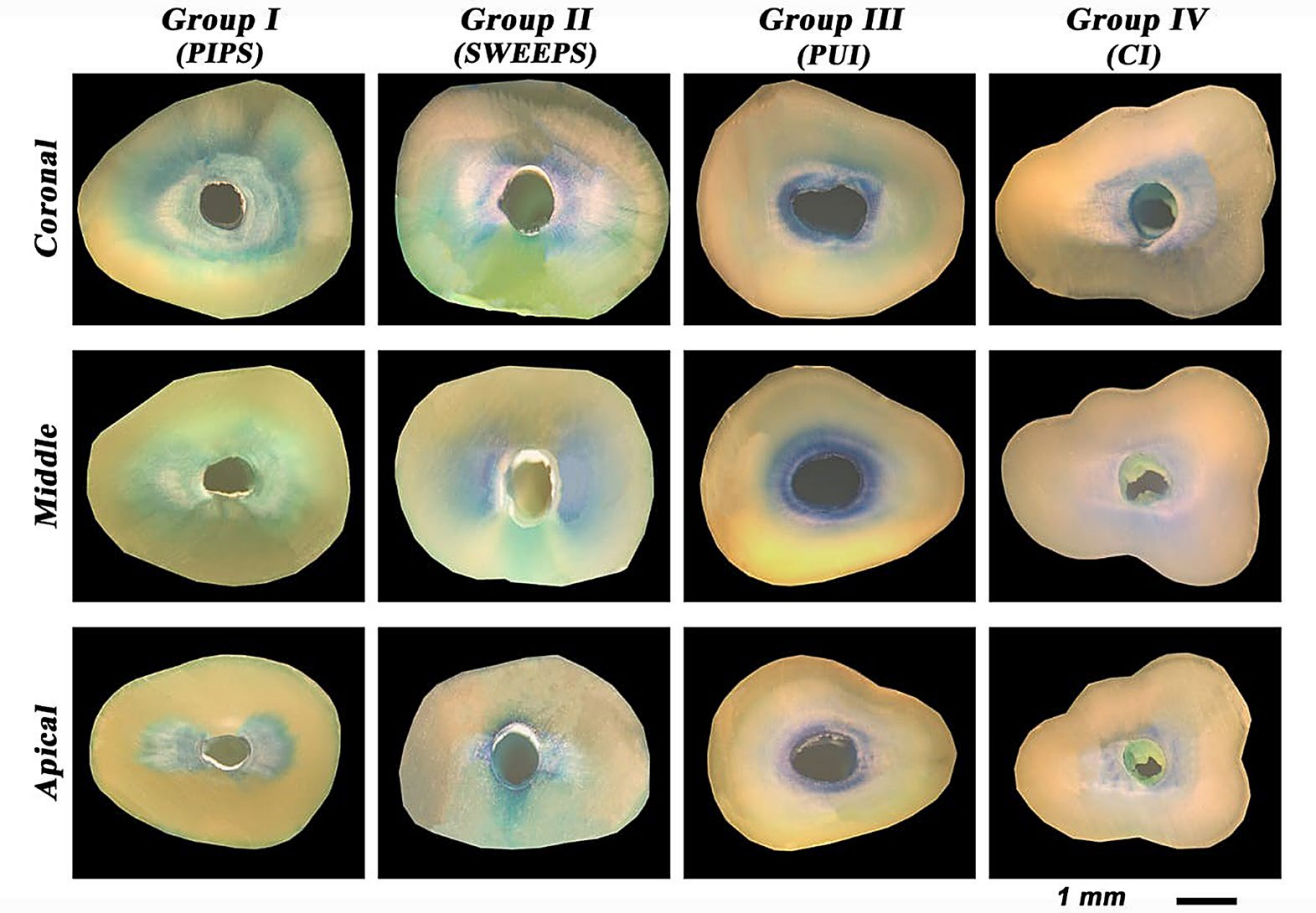
Representative images for each group at coronal, middle, and apical thirds showing MB dye penetration inside DTs

The results of penetration depth % and area % were presented in supplementary Table 1 and Fig. 1. Representative images for each group at the coronal, middle, and apical thirds are illustrated in Fig. 2.

Regarding the penetration depth %, all test groups (PIPS, SWEEPS, and PUI) showed statistically significant differences with the CI (control) group (*P* ≤ 0.05). However, no statistically significant differences where found between any of the test groups as shown in Table 1; Fig. 1. Differences between the test groups were negligible in coronal sections, where PIPS and SWEEPS groups showed nearly similar results compared to the PUI group. However, differences became obvious starting from the middle to the apical sections, where the PIPS and SWEEPS groups showed higher dye penetration than the PUI group as shown in supplementary Table 1 and Fig. 1.

Regarding the penetration area %, PIPS and SWEEPS groups showed significant differences with the CI (control) group (*P* ≤ 0.05), while no statistically significant differences were found between the test groupss as shown in Table 1; Fig. 1. The PIPS group showed the highest dye penetration area % in the coronal and apical sections, while SWEEPS showed the highest values in the middle section (supplementary Table 1).

**Table 1:** Pairwise comparisons among groups regarding penetration depth %, penetration area %, and Vickers microhardness

Group	Compared with	*P*		
		Penetration depth %	Penetration area %	Vickers microhardness
Group I	Group II	1.00	1.00	< 0.0001*
	Group III	0.16	0.26	< 0.0001*
	Group IV	< 0.0001*	0.001***	0.20
Group II	Group III	0.22	1.00	< 0.0001*
	Group IV	< 0.0001*	0.03****	0.0003*****
Group III	Group IV	0.05**	0.47	< 0.0001*

*Statistically significant difference (*P* ≤ 0.05)

Among groups, penetration depths/areas % were higher in the coronal thirds than in the Among groups, penetration depths/areas % were higher in the coronal thirds than in the middle thirds and similarly they were higher in the middle thirds than the apical thirds, with statistically significant differences between root thirds (*P* ≤ 0.05) as shown in Table 2. When comparing penetration depths/areas % within each group, both the PIPS and SWEEPS groups showed no statistically significant differences in penetration depth % between the coronal and middle thirds. However, there were significant differences between the coronal and apical thirds within these two groups. On the other hand, for both the PUI and CI groups there were no significant differences in their penetration depth % regardless of the thirds, as shown in Table 3. When considering the penetration area %, all groups showed statistically larger penetration areas in the coronal compared to the apical thirds. The PIPS and PUI groups additionally demonstrated significantly higher percentages of penetration areas for the coronal versus the middle thirds within each group. Contrarily, the SWEEPS group showed no statistically significant differences between the coronal and middle thirds, however, there were significant differences between the middle and apical thirds of this group, as shown in Table 3.

**Table 2:** Pairwise comparisons among thirds regarding penetration depth %, and penetration area %

Third	Compared with	*P*	
		Penetration depth %	Penetration area %
Coronal	Middle	0.04*	< 0.0001*
	Apical	< 0.0001*	< 0.0001*
Middle	Apical	0.04*	0.002*

*Statistically significant difference (*P* ≤ 0.05)

**Table 3:** Pairwise comparisons among thirds within the same group regarding penetration depth %, and penetration area %

Group	Third	Compared with	*P*	
			Penetration depth %	Penetration area %
Group I	Coronal	Middle	1.00	0.002*
		Apical	0.03*	< 0.0001*
	Middle	Apical	0.23	0.12
Group II	Coronal	Middle	1.00	0.12
		Apical	0.01*	< 0.0001*
	Middle	Apical	0.10	0.007*
Group III	Coronal	Middle	0.16	0.01*
		Apical	0.10	0.0009*
	Middle	Apical	1.00	1.00
Group IV	Coronal	Middle	0.94	0.19
		Apical	0.06	0.02*
	Middle	Apical	0.48	1.00

*Statistically significant difference (*P* ≤ 0.05)

Regarding dentin microhardness evaluation, SWEEPS group showed the highest Vickers hardness number (VHN) followed by the CI group, PIPS group and the PUI group, respectively. Statistically significant differences were found between SWEEPS group and all other groups. PIPS and CI (control group) showed similar values with statistically significant differences with the PUI group which showed the lowest VHN as shown in Table 1; Fig. 1.

## Discussion

The current study aimed to elucidate the effects of using recently introduced laser activation techniques; PIPS and SWEEPS on enhancing the penetration of irrigants as compared to the more established techniques of passive ultrasonic and conventional needle irrigation. These effects were also studied within the context of preserving microhardness while at the same time optimizing irrigant penetration.

Irrigation is important not only during the instrumentation step but also subsequently, as it helps flush out remaining microorganisms, tissue fragments, and dentinal debris [22]. It also helps avoid the accumulation of debris in the apical zone and the spread of infection to the periapical tissues [11]. Indeed, bacteria and debris hidden in the unprepared areas and inside DTs can be the source of persistent infection and cause failure of root canal therapy [23]. Furthermore, smear layer formed over canal surfaces during the root canal instrumentation may delay the action of endodontic irrigants which might affect the final outcome [23]. So many activation methods of irrigants have been propesed to improve the effect of irrigants and smear layer removal providing patent DTs permiting better penetration of irrigants inside inaccessible areas and DTs.

Laser-activated irrigation is a powerful endodontic procedure used to enhance canal cleanliness. Numerous studies have shown that PIPS activation using Er: YAG lasers has significantly higher efficacy compared to traditional methods. This is related to the rapid opto-dynamic phenomena during Super Short Pulse-assisted activation. This phenomena causes turbulent fluid movement within the whole root canal space, significantly improving the efficacy of the chemo-mechanical debridement [24, 25]. Moreover, the development SWEEPS, where the effective single-pulse PIPS irrigation is complemented with an additional dual-pulse shock wave technique. The SWEEPS modality depends on delivering a subsequent laser pulse into the liquid at an ideal time when the 1st bubble is in the final phase just before its collapse, The growth of the 2nd bubble exerts pressure on the collapsing initial bubble, accelerating its collapse and the collapse of secondary bubbles, resulting in primary and also secondary shock waves providing 3D streaming of the irrigant throughout the complex root canal system [3, 24].

The selected teeth in this study were single-rooted teeth with relatively oval canals as they can be challenging during chemo-mechanical preparation with risks of excessive dentin removal and higher chance of presence of untouched canal areas as compared to narrower and curved canals [26]. However, an advantage of this choice is comparability and reproducibility of irrigant penetration depth in different teeth due to the similar root canal anatomies. Furthermore, to standardize canal instrumentation and set the roots at a uniform working length; similar to previous studies [27, 28] all samples were standardized to 12 mm. The canal preparation to a size 30\6% taper was selected to enable better penetration of the irrigants and might be considered as minimum canal preparation size allowing adequate irrigation penetration, removal of debris and smear layer in the apical third as mentioned by previous studies [29–31].

In the present study, MB dye was used to show penetration depth/area as an indirect indicator for the ingress of NaOCl as in previous studies [3, 15]. Penetration depth of MB dye in micrometers was initially measured for all sections in all groups, after that the penetration depth % was calculated by dividing the penetration depth by dentin thickness. Dye penetration as a percentage of the section radius would be of greater importance especially in narrower roots, because the percentage of dye penetration gives a more standardized and comparable metric by providing a proportional measure that accounts for anatomical variations, making it more relevant for comparing samples in relation to the root’s structural dimensions.

Results of the present study confirmed the superior effects of PUI compared to CI and interestingly were comparable to both PIPS and SWEEPS. Ultrasonic activation has a high driving frequency of ultrasound producing powers of cavitation and acoustic streaming which leads to a better flow of irrigants, resulting in more effective delivery of irrigant inside the root canals [3, 32].

On the other hand, in the present study, SWEEPS exhibited no superior efficacy over PIPS where both showed nearly equal dye penetration depth and area % inside DTs. That was in line with Galler KM et al. [3] who examined penetration depths of irrigation solutions with different activation methods, and lower penetration depths were found in the SWEEPS group compared with PIPS. Moreover, our results where correlated with another study by Kosarieh E et al. [15] which tested the MB dye penetration depth after root canal preparation using PIPS and SWEEPS methods, showing no statistically significant difference between both groups with higher values of dye penetration in the PIPS group. These results might be attributed to the fact that in the SWEEPS mode, the pulse pairs and subsequent bubbles might have caused a counter-current impeding irrigant flow within the root canal.

The smaller but still considerable penetration of dye in the control group might be justified by the preparation size of the canals in combination with thorough irrigation using NaOCL and EDTA solutions where it appears that the smear layer reduces but not fully prevents ingress of irrigants into the DTs [3]. Moreover, the use of EDTA, to remove the smear layer and the subsequent rinse with NaOCl which would now penetrate more easily into the DTs, has been advocated [33]. These factors possibly aided the penetration depths in the control along with the test groups in the coronal, middle and apical sections.

Among groups, the penetration depth/area % of MB dyes in the coronal third was significantly higher than the middle and apical thirds. Our results were consistent with a study by Akcay M. et al. [34] and Karaoğlu G. et al. [35]. Lo Giudice G et al. [36], demonstrated that the number of DTs decreases from the coronal to the apical direction. This can justify the lower penetration depth from coronal to apical sections in our study. Both PIPS and SWEEPS groups showed no statistically significant differences between the coronal and middle thirds yet, significant differences between the coronal and apical thirds within these groups were found. This may indicate that their ability to penetrate deeper into dentin is preserved beyond the middle third of the canal, however, their effects become milder in the apical thirds. On the other hand, for both the PUI and CI groups there were no significant differences in their penetration depth regardless of the thirds indicating that these methods of irrigation did not enhance the ability of the irrigant to penetrate deeper into DTs. When considering the penetration area percentage, all groups showed statistically larger penetration areas in the coronal compared to the apical thirds. The PIPS and PUI groups additionally demonstrated significantly higher percentages of penetration areas for the coronal versus the middle thirds within each group. Contrarily, the SWEEPS group showed no statistically significant differences between the coronal and middle thirds. However, there were significant differences between the middle and apical thirds of this group. This again confirms the ability of SWEEPS to enhance irrigation homogeneously and effectively in the coronal and middle thirds; however, its efficacy is compromised in the apical thirds of the canals. It is noteworthy to mention that most studies [3, 15] focus on measuring penetration depth rather than the area percentage of penetration which was additionally performed in the current study. While depth may be more important from a microbiological perspective to efficiently attack deep seated bacterial biofilms particularly in long standing infections, the entire surface area exposed to the irrigant may be of equal importance particularly when considering the development and evolution of novel irrigants with potential substantivity to cover larger surface areas.

During irrigant activation, irrigating solutions simultaneously could cause changes in the physical properties of dentin, which might decrease its microhardness. Therefore, dentin microhardness was subsequently assessed in the present study. The microhardness test is a simple and commonly used test to study fine-scale changes in the dentin hardness. Vicker’s hardness test has been considered a suitable and a practical one, to estimate the changes in the surface and as well as deeper hard tissues’ structures [13, 22]. In our study, 3 points in each sample were selected for measuring the micro-hardness, in order to minimize structural variations. Microhardness testing was done only for the coronal third of the root because the coronal third needs requires a conservative approach as it is the most affected third by irrigants as previuosly shown in several studies [37, 38]. Moreover, the preservation of cervical dentin could in turn enhance the resistance of the tooth to fracture under masticatory loads [39]. To standardize the measurements, indentations were made on the dentin surface at approximately 200 μm from the dentin-canal interface [21]. It is worth mentioning that in this study activation was only for NaOCl and not EDTA solution to decrease the contact time of EDTA which might cause erosion of dentin which would change its viscoelastic properties and hence affect its microhardness [40, 41].

The results of this study showed that the SWEEPS activation method provided the higher microhardness values with statistically significant differences compared to other groups, which might be attributed to the high-speed and pressure of irrigant flow compared to other methods of activation [42]. This might have decreased the contact time of irrigant with the canal walls which could have decreased the mineral loss of dentin. To the best of the authors’ knowledge, this is the first study to evaluate the effect of SWEEPS activation on dentin microhardness.

The PIPS activation method did not cause an additional decrease in dentin microhardness compared to the CI (control group), which could be attributed to the fact that PIPS activation caused minimal change in mineral content as previously mentioned in a study by Topçuoğlu HS [43]. Moreover, this may be due to the absence of thermal damage of PIPS treated samples. PIPS has been found to cause a minimal temperature rise (< 1.5 °C) and cause minimal surface alternations of the root surface [44]. Our results were in line with Akbulut M. et al. [14] and Shi L et al. [11] where no statistically significant differences were found between PIPS activation and CI regarding their effect on dentin microhardness, showing that the effect on the microhardness values is mainly affected by the type of irrigant itself not by the activation method.

The PUI activation method showed the least dentin microhardness values compared to the other groups. This result was in line with Arul B. et al. [45], Sandra N. et al. [46] and Irina T. et al. [40]. It could be suspected that the PUI acoustic streaming might have adverse effects over the bio-mechanical characteristics of root dentin [40, 46]. Also, this effect might be attributed to the increase in irrigant temperature when activated by PUI which was stated by many previous studies [47, 48]. This rise of irrigant temperature may cause dissolution of collagen fibers which might affect microhardness [49].

This study still has some limitations. Firstly, no activation was done for MB dye which could have demonstrated more irrigant penetration into the DTs. However, activation of methylene blue could have added a confounding variable which might influence the results as dye penetration in this study was used as an indicator of canal wall cleanliness, focusing on the effectiveness of the irrigation techniques rather than evaluating the dye’s behavior under activation as previously done by Kosarieh E. et al. [15]. Secondly, regarding the microhardness analysis, another limitation is that it was not possible to obtain baseline values before activation methods of the same samples. Alternatively, we utilized a control group where CI were used without irrigant activation as previously done in a previous study [46] for better simulation of the clinical situation instead of teeth sectioning and immersion technique adopted by other studies [50, 51].

## Conclusions

Based on the findings of this study:

1) Laser irrigant activation using PIPS and SWEEPS is comparable to PUI but more effective than CI in dye penetration into dentinal tubules, offering more advantages as they do not depend on the insertion depth of a file or probe, unlike other tested techniques.

2) The SWEEPS method preserves dentin microhardness, supporting the long-term prognosis of root canal-treated teeth.

## Electronic supplementary material

Below is the link to the electronic supplementary material.

Supplementary Material 1

## Data Availability

Data is provided within the manuscript or supplementary information files.The datasets used and/or analysed during the current study are available from the corresponding author on reasonable request.

## Abbreviations

MB: Methylene Blue
PIPS: Photon-induced photoacoustic streaming
SWEEPS: Shockwave enhanced emission photoacoustic streaming
PUI: Passive ultrasonic Irrigation
CI: Conventional irrigation
DTs: Dentinal tubules
NaOCl: Sodium Hypochlorite
EDTA: Ethylenediaminetetraacetic acid
WL: Working length
