# Supplementary material for: In vitro evaluation of dye penetration and dentin microhardness after laser irradiation using photon-induced photoacoustic streaming and shock wave enhanced emission photoacoustic streaming tips compared to ultrasonic activation
Source: Lasers Med Sci. 2025 Jan 31;40(1):57. doi: 10.1007/s10103-025-04310-4 (PMC11785666; doi:10.1007/s10103-025-04310-4)
Supplement: Supplementary file 1 — Supplementary Material 1 [file 10103_2025_4310_MOESM1_ESM.docx]

**Supplementary table 1: Descriptive values of** **penetration depth %, penetration area %, and Vickers microhardness for the four study groups**

| Test | Third | Group I  (n=10) | Group II  (n=10) | Group III  (n=10) | Group IV  (n=10) |
| --- | --- | --- | --- | --- | --- |
| Penetration depth % | Coronal  x̄ (SD) | 59.36  (17.24) | 58.30  (7.63) | 55.35  (13.17) | 38.02  (10.48) |
|  | Middle  x̄ (SD) | 53.45  (13.90) | 53.44  (8.50) | 36.51  (25.65) | 31.85  (12.83) |
|  | Apical  x̄ (SD) | 41.17  (13.51) | 40.54  (19.18) | 34.40  (22.14) | 23.19  (16.22) |
| Penetration area % | Coronal  x̄ (SD) | 43.90  (9.56) | 36.23  (9.71) | 36.41  (10.82) | 23.86  (7.49) |
|  | Middle  x̄ (SD) | 25.35  (12.23) | 27.08  (8.41) | 18.19  (14.73) | 16.24  (9.83) |
|  | Apical  x̄ (SD) | 14.79  (10.64) | 12.84  (10.05) | 12.66  (12.74) | 12.51  (9.02) |
| Vickers microhardness | Coronal  x̄ (SD) | 72.96  (2.63) | 82.20  (3.91) | 56.57  (3.15) | 75.93  (2.04) |

SD, standard deviation; x̄, mean

**Supplementary table 2: Two-way ANOVA of penetration depth %, and penetration area % for the four study groups**

| Test | Variables | Mean square | F test | *P* | Ƞp^2^ |
| --- | --- | --- | --- | --- | --- |
| Penetration depth % | Group | 2713.78 | 10.74 | <0.0001* | 0.23 |
|  | Third | 3215.67 | 12.73 | <0.0001* | 0.19 |
|  | Interaction | 124.54 | 0.49 | 0.81 | 0.03 |
| Penetration area % | Group | 605.42 | 5.38 | 0.002* | 0.13 |
|  | Third | 4875.07 | 43.31 | <0.0001* | 0.45 |
|  | Interaction | 188.55 | 1.67 | 0.13 | 0.09 |

Ƞp^2^, partial eta squared

*Statistically significant difference (P≤0.05)

**Supplementary table 3: One-way ANOVA for Vickers microhardness test for study groups**

| Test | Mean square | F test | *P* |
| --- | --- | --- | --- |
| Vickers microhardness | 1195.11 | 131.84 | <0.0001* |

*Statistically significant difference (*P*≤0.05)

**Supplementary table 4: Pairwise comparisons among thirds regarding penetration depth %, and penetration area %.**

| Third | Compared with | *P* | |
| --- | --- | --- | --- |
|  |  | Penetration depth % | Penetration  area % |
| Coronal | Middle | 0.04* | <0.0001* |
|  | Apical | <0.0001* | <0.0001* |
| Middle | Apical | 0.04* | 0.002* |

*Statistically significant difference (*P*≤0.05)

**Supplementary table 5: One-way ANOVA of penetration depth %, and penetration area % among thirds within the same group**

| Test | Variables | Mean square | F test | *P* |
| --- | --- | --- | --- | --- |
| Penetration depth % | Group I | 861.64 | 3.84 | 0.03* |
|  | Group II | 841.79 | 5.07 | 0.01* |
|  | Group III | 1331.33 | 3.02 | 0.07 |
|  | Group IV | 554.53 | 3.09 | 0.06 |
| Penetration area % | Group I | 2172.35 | 18.40 | <0.0001* |
|  | Group II | 1389.40 | 15.67 | <0.0001* |
|  | Group III | 1544.79 | 9.33 | 0.0008* |
|  | Group IV | 334.19 | 4.28 | 0.02* |

*Statistically significant difference (*P*≤0.05)
